# Young and Middle-Aged Schoolteachers Differ in the Neural Correlates of Memory Encoding and Cognitive Fatigue: A Functional MRI Study

**DOI:** 10.3389/fnhum.2016.00148

**Published:** 2016-04-07

**Authors:** Elissa B. Klaassen, Sarah Plukaard, Elisabeth A. T. Evers, Renate H. M. de Groot, Walter H. Backes, Dick J. Veltman, Jelle Jolles

**Affiliations:** ^1^School for Mental Health and Neuroscience (MHeNS), Department of Psychiatry and Neuropsychology, Maastricht UniversityMaastricht, Netherlands; ^2^Faculty of Behavioral and Movement Sciences, VU University AmsterdamAmsterdam, Netherlands; ^3^Faculty of Psychology and Neuroscience, Maastricht UniversityMaastricht, Netherlands; ^4^Centre for Learning Sciences and Technologies (CELSTEC), Open UniversityHeerlen, Netherlands; ^5^Department of Radiology, Maastricht University Medical CentreMaastricht, Netherlands; ^6^Department of Psychiatry, VU University Medical Centre, and Neuroscience Campus AmsterdamAmsterdam, Netherlands

**Keywords:** episodic memory, schoolteachers, aging, mental fatigue, fMRI, middle age

## Abstract

This investigation was inspired by growing evidence that middle-aged persons in a cognitively demanding profession might be characterized by subtle cognitive fatigue. We studied young and middle-aged male schoolteachers. They were compared in a study with functional magnetic resonance imaging to evaluate differences during successful memory encoding. The schoolteachers were additionally subjected to an induced fatigue condition involving the sustained performance of cognitively demanding tasks and to a control condition. Results showed age-related brain activation differences underlying behavioral performance including: (1) greater activation in middle-aged vs. young teachers in bilateral prefrontal cortex (PFC) areas; and (2) differential fatigue effects in the left anterior cingulate cortex (ACC) depending on age group. Middle-aged schoolteachers showed decreased ACC activation in the fatigue compared to the control condition, whereas no change in activation was found in young teachers. Findings demonstrate age effects in these middle-aged subjects that are typically found in older adults, specifically in PFC over-activation. Findings also indicate that already in middle age cognitive aging may be associated with greater resource depletion following sustained task performance. The findings underscore the notion that persons in a cognitively demanding profession can experience subtle age effects, which are evident on fMRI and which impact daily functioning. Possible practical implications for middle-aged schoolteachers are discussed.

## Introduction

Memory function declines across the adult lifespan (e.g., Park et al., 2002; Myerson et al., 2003; Salthouse, 2003; Bopp and Verhaeghen, 2005; van der Elst et al., 2005; Soei and Daum, 2008; van der Elst and Jolles, 2012), with episodic memory thought to show the greatest decline (Reuter-Lorenz and Park, 2010; but see Rönnlund et al., 2005). The majority of aging studies focus on differences between young and old adults aged 60+. Although aging effects can also be expected in middle age—between 40 and 60 years—little is known about the nature and impact of memory decline at this stage of life (e.g., Burgmans et al., 2010, 2011). A recent fMRI experiment focused on negative subsequent memory and task-negative effects (de Chastelaine et al., 2011). These authors showed that suppression of activation within the default mode network is attenuated in middle-aged compared to young individuals during an encoding task. This suggests that middle-aged individuals become less competent at reallocating internally directed processes to those necessary for accomplishing the task. However, there were no clear indications for changes in negative subsequent memory in middle age. Other recent studies (e.g., Park et al., 2013; Klaassen et al., 2014; Cansino et al., 2015) are consistent with the notion that there are subtle effects on memory-related functions in middle age, which are accompanied by fMRI changes.

The relative lack of studies that include middle-aged adults can be attributed to the subtle nature of the age-related decline in memory performance in this age group. Yet, the subtlety of age-related behavioral changes may belie more extensive brain activation changes. Studies using functional magnetic resonance imaging (fMRI) have demonstrated significant brain activation differences between young and old adults (Morcom et al., 2003; Gutchess et al., 2005; Dennis et al., 2007; Park and Reuter-Lorenz, 2009). In their studies, Gutchess and Morcom also found differences in memory performance in terms of more false alarms and changes in recognition memory. The findings are taken to imply that middle-aged subjects are characterized by subtle neurocognitive changes. We have suggested that the middle-aged have to invest more cognitive effort in order to keep their performance on the level of younger adults (e.g., Jacobs et al., 2013; Klaassen et al., 2013, 2014). This increased effort is reflected in the changes in brain activation that have been found in these studies. This suggests that middle-aged and older adults will probably have a problem with sustaining their performance on demanding tasks for a more prolonged period of time and/or in a situation of increased workload. This is what the present study investigates. We used fMRI to examine activation differences between young and middle-aged teachers during episodic memory encoding. The subjects were tested after a long workday in a control condition and in a “fatigue” condition. The fatigue condition required the subjects to sustain their performance on cognitively demanding tasks.

The current study was inspired by our earlier studies of cognitive performance in middle-aged schoolteachers (van der Elst et al., 2012) and other healthy subjects (Burgmans et al., 2011). Unlike older adults who are—generally—retired, middle-aged adults commonly work fulltime. They are consequently faced with the challenge of maintaining a high level of performance throughout the workday. Because of the subtle effects of cognitive aging mentioned above, it can be expected that performance of middle-aged subjects may be especially compromised because of their challenging job. Furthermore, the impact of aging on memory performance and other cognitive functions in middle age is probably particularly relevant for persons who have an occupation where workday cognitive demands are high. Schoolteachers are a good example of an occupational group who have to sustain a high performance level and stay alert from early in the morning to late in the afternoon. This is said to be one of the reasons for an increased prevalence of burnout in middle-aged schoolteachers (van der Elst et al., 2012). Schoolteachers are therefore a very relevant target group for evaluating the thesis that middle-aged subjects may be characterized by brain activation differences in comparison to younger subjects when they have to perform at a high level during a workday with high cognitive demands. The first aim of the study is to find out whether there are differences between young and old schoolteachers in brain activation and cognitive performance. A second aim is to evaluate the possible additional effect of an intervention that increases cognitive fatigue. Male schoolteachers were selected for this study in order to increase group homogeneity in view of the known sex differences in cognitive performance (van der Elst et al., 2005, 2006a,b) and in functional neuroanatomy (Cahill, 2006). The selection of male subjects and the use of a homogeneous group increased the possibility for the detection of age-related differences.

Young and middle-aged teachers were compared in terms of successful memory encoding performance and brain activation using the subsequent memory paradigm (Paller and Wagner, 2002). In this paradigm, items studied during an encoding task are classified according to performance on a subsequent recognition task; items that were subsequently remembered are contrasted with items that were subsequently forgotten. Previous studies using the successful encoding paradigm have demonstrated activation differences including increased or more bilateral (Morcom et al., 2003) prefrontal cortex (PFC) recruitment. These findings were sometimes accompanied by reduced (left or bilateral) medial temporal lobe (MTL) activation in old compared to young adults (Gutchess et al., 2005). These activation changes are thought to underlie the fact that performance at the behavioral level was similar for the two groups. These findings are commonly suggested to reflect the action of neural compensation processes (Cabeza et al., 2002; Dennis et al., 2007; Park and Reuter-Lorenz, 2009).

With respect to activation changes, both increased and/or more dispersed PFC activation have been found, particularly more bilateral. These changes are taken to indicate that there is increased recruitment of neural resources. Similarly, on the behavioral level, the subject has to invest greater cognitive effort. This greater cognitive effort—supported by increased recruitment of neural resources, is thought to facilitate the maintenance of a higher level of behavioral performance. The interpretation of increased and/or more dispersed neural activation depends on whether performance is maintained, increased or decreased. When neural increases are accompanied by performance declines, the more plausible interpretation would be impairment, e.g., inefficient processing or neural dedifferentiation (Reuter-Lorenz and Lustig, 2005). Notably, the notion of age-related reorganization could be challenged since the evidence is mainly based upon cross-sectional designs, as discussed by Nyberg et al. (2010). With the present study, we aimed to determine whether middle-aged adults similarly rely on the increased recruitment of neural resources to maintain a similar level of performance to young adults. This investigation was inspired by experiential reports from teachers indicating that middle-aged teachers feel that some aspects of teaching, in particular learning and implementing new educational practices, are more demanding and cost them more time and effort, than their younger colleagues (van der Elst et al., 2012).

In demanding occupations, such as teaching, a typical workday commonly involves prolonged periods of demanding cognitive activity. The job of a schoolteacher typically requires the subject to switch attention between the presentation and elaboration of content (educational material) and paying attention to the students in the classroom and their behavior. Teachers thus constantly rely on working memory and attentional control functions of the brain. Moreover, they have to sustain their performance from early in the morning through their workday until the school bell rings for the last time. It could thus very well be that middle-aged teachers experience more problems in sustaining their performance level, and—if they succeed—this may require continuous effort and thus give rise to cognitive fatigue. It is possible to test this notion by experimental intervention, and that is what we did. In the present study, we not only compared middle-aged to young teachers at a baseline level (control condition). We also compared the effect of an *additional* intervention in both age groups. This intervention—the so called “induced fatigue condition” — involved the sustained performance of cognitively demanding tasks that engage working memory and attentional control.

We consider the teachers used in the present study to be a model for other demanding occupations that require a lot of social interactions with a high workload and responsibility, and frequent attentional shifting for an extended period of the workday. The necessity to engage neural compensation processes in middle age may also influence the effects of such periods of demanding cognitive task performance. Specifically, the capacity for middle-aged teachers—and other demanding occupations—to sustain a high level of performance may be constrained by the limited nature of neural resources. Hence, the limited capacity of age-related neural compensation processes (Park and Reuter-Lorenz, 2009) can be expected to underlie performance declines or increases in cognitive fatigue and behavioral side-effects. Several fMRI studies have provided support for the limited capacity of neural compensation in older subjects. Increased activation underlying equivalent task performance was found in older compared to younger adults when task demands were low. However, in those cases when the task demands were high, the activation was relatively decreased and accompanied by performance declines in older adults (Cabeza et al., 2000; Cappell et al., 2010; Schneider-Garces et al., 2010). The point at which resources supporting neural compensation are exhausted and performance declines ensue has been termed the “crunch” point by Reuter-Lorenz and Cappell (2008) in their Compensation-Related Utilization of Neural Circuits Hypothesis (CRUNCH). Notably, although neural compensation can involve a wide range of brain areas for a wide variety of tasks, it generally involves areas in the PFC in association with executive functions or (working) memory tasks (Reuter-Lorenz and Cappell, 2008).

The sustained performance of cognitive tasks requiring a high level of cognitive effort (DeLuca, 2005) is thought to result in a state of induced cognitive fatigue as a result of the temporary depletion of limited cognitive resources (Smit et al., 2004; Persson et al., 2007). Aspects of this induced fatigue state include an increase in subjective fatigue accompanied by cognitive performance decrements and a decreased amplitude of event-related potential components (e.g., Lorist et al., 2000, 2005, 2009; van der Linden et al., 2003; Boksem et al., 2006; Lorist, 2008; Kato et al., 2009). We hypothesized that the induced fatigue condition would result in a depletion of cognitive resources, evident as a decrease in brain activation and an associated performance decrement in both age groups. Furthermore, we hypothesized that decreased activation would be particularly evident in brain areas associated with cognitive control in memory such as the left PFC (Badre and Wagner, 2007; Blumenfeld and Ranganath, 2007). Similar findings were expected with respect to cognitive control in general (such as the anterior cingulate cortex, ACC). This is because several studies have shown that higher-level cognitive control functions are particularly sensitive to detrimental induced cognitive fatigue effects (Lorist et al., 2000, 2005; van der Linden et al., 2003). Finally, we expected to find that the age-related increase in the recruitment of neural resources in middle-aged adults would be associated with greater subsequent exhaustion of cognitive resources. This expectation was based upon the above-mentioned limitations of neural resources by fatigue as well as age. Together with decreases in brain activation, we expected greater performance decrements following the sustained performance of cognitively demanding tasks. In other words, we expected the effects of our fatigue manipulation on neural activation to be greater in the middle-aged compared to the young teachers.

## Materials and Methods

### Participants

Healthy, right-handed, young (14 participants aged 25–35 years) and middle-aged (18 participants aged 50–61 years) Dutch male schoolteachers (working fulltime) were recruited via advertisements placed in school bulletins, flyers distributed at schools, or short information sessions for teachers. Volunteers were screened and those who suffered significant past or present physical or psychiatric illness, received medication (other than antihypertensives in two middle-aged adults), reported alcohol or drug abuse, or had MRI contraindications, were not included.

Three participating middle-aged teachers were excluded from the analysis due to intervening panic in the scanner, incorrect task execution, and a technical error during the encoding task, leaving 14 young (mean age = 30.6, standard deviation = 3.2, range = 25–35) and 15 middle-aged (mean age = 55.4, standard deviation = 3.9, range = 50–61) teachers for group analysis.

We restricted our sample to males, as our study concerned a less extreme age group comparison than most aging studies. Testing males only increased sensitivity for the detection of age-related differences by increasing group homogeneity (sex differences are relatively large in terms of functional neuroanatomy: Cahill, 2006; and cognitive performance: van der Elst et al., 2005, 2006a,b) and minimizing variance between repeated measures, as females show increased fluctuation of cognition, mood and fatigue in relation to the menstrual cycle (Farage et al., 2008). The study was approved by the local medical ethical committee at Maastricht University Academic Hospital. Volunteers gave informed consent prior to their (paid) participation.

### Procedure

Young and middle-aged teachers were compared in a to-the-tester-blind, randomized crossover study design. Participants completed a training session (in the week prior to the first test session) and two test sessions (administered on two consecutive weekends with both sessions starting at either 0900, 1100 or 1300 h). During the training session, participants completed a battery of neuropsychological tests and practiced the fMRI tasks in a dummy MRI scanner to become familiarized with the scanning environment and minimize practice effects.

During the test sessions, participants spent the first 1.5 h completing either the control or the induced fatigue manipulation outside the MRI scanner. They then entered the scanner where they were scanned during the memory encoding task. An additional cognitive task (involving letters only, making interference with the present task unlikely) and a resting state measure were also completed in the scanner, the results of which will be reported elsewhere. Each participant was therefore tested twice and the order of control or fatigue condition administration was randomized. Subjective fatigue levels were measured throughout the test sessions via a subjective rating scale. The researcher operating the scanner and providing instructions to participants during scanning was blind to the manipulation condition the participants had just completed.

### Neuropsychological Tests

A battery of standardized neuropsychological tests was administered to assess: (a) memory processes investigated in our fMRI tasks; (b) other cognitive functions known to decline, increase or remain stable with age; and (c) the intelligence characteristics of the sample. The visual verbal Word Learning Test (WLT; van der Elst et al., 2005) was administered as a measure of immediate and delayed memory recall and recognition, whereas the digit span (forward and backward) was administered to test short-term/working memory capacity (Lezak et al., 2004). General cognitive functions were tested using the Letter Digit Substitution Test (LDST; van der Elst et al., 2006a) and the letter verbal fluency test (van der Elst et al., 2006b). Finally, the Dutch version of the National Adult Reading test (Nelson, 1991; Schmand et al., 1991) was administered as a measure of mental ability (intelligence) in adults based on vocabulary.

### fMRI Encoding and Recognition Tasks

During the fMRI encoding (6 min) and recognition (16 min) tasks, 100 words were presented one by one on the screen in pseudorandom order such that the proximity of highly semantically or phonetically similar words was minimized. Words were divided equally into four semantic categories: food (F), animals (A), utensils/tools (U) and landscape features (L). Two different word lists were constructed and used to create two versions of the task, which were then randomized in the repeated measures design. The two word lists were matched with regard to factors such as word length, the number of syllables in each word and frequency of use in everyday language.

During encoding, participants were instructed to indicate the category to which a word belonged by pressing the appropriate button, using the left- and right-hand middle and index fingers. The categories were displayed at the bottom of the screen as each word was presented (as: F A U L). Participants were aware that they would subsequently be required to remember the encoding task words.

During recognition, the same 100 “old” words were presented, plus an additional 100 “new” words. Participants were instructed to indicate with a button-press response whether they judged each word to be old or new, and how confident they were about this judgment. Response options therefore included: definitely old, probably old, probably new, and definitely new (displayed at the bottom of the screen as: Old 1 2 3 4 New). Encoding and recognition tasks were separated by a period of about 15 min, during which participants completed an unrelated task.

Encoding and recognition task words were presented in blocks of eight stimuli followed by three null trials (consisting of a fixation point). Words were displayed in the center of the screen for 2500 ms, followed by a jittered inter-trial interval (ITI; 500–1250 ms) in accordance with Dennis and Cabeza (2011). The jitter was introduced to reduce the correlation between the blood oxygenation level-dependent (BOLD) response and previous trials given the short ITI. In the training session, the tasks were practiced with 200 words from the categories sport, country, city and occupation.

### Control and Fatigue Manipulations

In the fatigue manipulation, participants performed the following tasks: 2 and 3-N-back task (3 × 10 min), Stroop task with additional auditory interference (with auditory noise; 2 × 10 min), mental arithmetic (20 min), and brain teasers/puzzles (20 min). These tasks were selected for the high demands they place on a range of executive functions also subsequently involved in the scanning tasks. During the control manipulation, participants watched a documentary style DVD and/or read a magazine (e.g., the National Geographic) at their leisure.

### Subjective Fatigue Ratings

The fatigue subscale of the Dutch visual analog scale (VAS: with scores ranging from 0 to 100) short version of the Profile of Mood States (POMS) was administered (Wald and Mellenbergh, 1990) at three time points: before the manipulation (time 0), between the manipulation and MRI scanning (time 1), and after scanning (time 2). The POMS fatigue subscale (consisting of six items) is a recommended measure of subjective fatigue in investigations that are short in duration (e.g., a few hours; O’Connor, 2006), and was administered to determine the effect of the fatigue manipulation on the “mood of fatigue”. Mood of fatigue refers to “feelings of having a reduced capacity to complete mental or physical activities” (O’Connor, 2004, pp S7).

To assess longer-term feelings of fatigue in relation to the teaching profession and age-related memory and learning decline, we administered the following VAS questions during the training session: “Do you have a fatiguing job?”, “Are your daily activities fatiguing?”, and “Does fatigue interfere with your ability to learn new things?”.

### MRI Data Acquisition

Scans were made in a 3 Tesla Philips whole body scanner (Philips Achieva, Philips Medical Systems, Best, Netherlands). A body coil was used for RF transmission with an 8-element SENSE head coil for signal detection. During the encoding task approximately 180 echo-planar imaging (EPI) scans were made (TR = 2.0 s, TE = 35 ms, number of slices = 32, image matrix = 64 × 64, voxel size = 4 mm × 4 mm × 3.5 mm). A T1-weighted anatomical scan was also acquired for anatomical reference and coregistration of the two test sessions (image matrix = 256 × 256, number of slices = 150, voxel size = 1 mm × 1 mm × 1 mm).

### MRI Data Analysis

SPM8 (Statistical Parametric Mapping: Wellcome Trust Centre for Neuroimaging, Institute of Neurology, University College London) was used to preprocess and analyze the fMRI data. Preprocessing steps included: slice time correction, realign and unwarp, coregistration (session 2 scans were coregistered to session 1 scans), spatial normalization (Montreal Neurological Institute (MNI) space, EPI template), and smoothing (with a Gaussian kernel with a full-width at half maximum (FWHM) of 8 mm). Functional MRI data was analyzed in an event-related analysis in which activity related to the various event types was modeled by convolving a vector of the onset times with the canonical hemodynamic response function within the context of the general linear model. Incorrectly categorized words during the encoding task were modeled separately as errors, whereas correctly categorized words were modeled based on subsequent memory performance during the recognition task as: (1) subsequently recognized (with high confidence); and (2) subsequently forgotten (including subsequently recognized with low confidence and subsequent misses; Morcom et al., 2003; Duverne et al., 2009). In addition, motion parameters were included to correct for motion-related activation.

The main effect of age group and fatigue condition, and the interaction between age group and fatigue condition were investigated by entering individual task contrasts (contrasting subsequently recognized with subsequently forgotten events) into a second level two (control vs. fatigue condition) by two (young vs. middle-aged teachers) Full Factorial model. In case of significant interaction effects, these contrasts were used as an exclusive mask when testing for main effects to ensure that areas showing a significant interaction were not included in our main effects analyses. Successful encoding was operationalized by the contrast: subsequent recognition > subsequent forgetting. Task-related activation (activation across the control and fatigue conditions associated with the successful encoding contrast in both age groups) was examined at *p*_(uncorrected)_ < 0.001, with a voxel threshold > 10, with reported activation clusters significant at the cluster-level at *p*_(Family−wise error corrected; FWE)_ < 0.05. We used the random field theory (RFT) approach provided by SPM in which the smoothness of the statistical map is estimated followed by calculation of the alpha level based on RFT.

Furthermore, given findings from previous studies, we focused on age and fatigue condition effects in the PFC and MTL, based on Anatomical Automatic Labeling (AAL) regions defined according to the SPM8 WFU pickatlas (Tzourio-Mazoyer et al., 2002; Maldjian et al., 2003, 2004), using the small volume correction option in SPM8 to construct 12 mm radius spheres centered around the peak voxels identified for the main effect of task, which is orthogonal with respect to group and condition interaction effects (Kriegeskorte et al., 2009). Within these small volumes, age and fatigue effects were considered significant at *p*_(FWE)_ < 0.05. *Post hoc*, significant interaction effects were followed up by correlation analyses to examine associations between task performance and regional activation in each group, using the MarsBar toolbox to extract the mean signal from each region of interest (ROI). This was done to evaluate whether activation in these areas would for example indicate performance facilitation or rather interference. The main analysis was repeated using a contrast of subsequent recognition > subsequent misses (excluding low confidence subsequent recognition from the contrast); however, this did not change the main pattern of results.

## Results

### Neuropsychological Data

Scores on the neuropsychological tests are shown in Table 1. Independent samples *t*-tests showed a trend for lower scores by middle-aged compared to young adults on the WLT immediate free recall subtest. Differences between the two age groups were not apparent on the WLT delayed free recall subtest, the WLT delayed cued recognition subtest, the Digit span test or the LDST. Scores were significantly higher in middle-aged compared to young teachers on the Dutch adult reading test and the letter fluency test, suggesting higher verbal intelligence in the middle-aged group despite matched education level and occupation type.

**Table 1:** **Neuropsychological test scores**.

	Young			Middle age		
	Mean	SD	Range	Mean	SD	Range	*t*	*p*
WLT immediate free recall	12.5	1.9	9–15	10.9	2.3	7–14	2.03	0.053
WLT delayed free recall	10.9	3.2	5–15	9.9	2.4	6–14	0.96	0.298
WLT delayed cued recall	14.4	0.8	13–15	14.3	1.1	12–15	0.28	0.783
Digit span	17.5	2.6	14–24	16.8	4.0	12–24	0.55	0.584
LDST	57.0	8.1	42–70	52.7	7.0	37–66	1.53	0.137
Letter fluency*	13.6	3.0	8–18	17.2	4.7	9–23	2.03	0.021
Dutch adult reading test**	80.9	7.8	65–90	88.9	4.7	82–96	3.35	0.003

Significant age group differences: *p < 0.05; **p < 0.01. Abbreviations: WLT, visual verbal word learning test; LDST, letter digit substitution test.

### Subjective Fatigue Ratings

**Figure 1:**
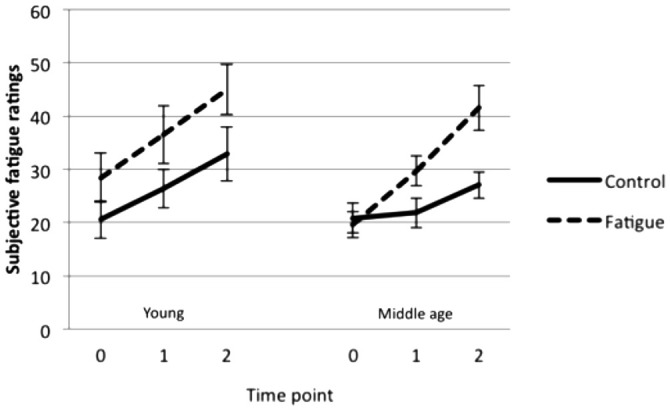
**Mean subjective ratings (± SE) before the manipulation (time 0), between the manipulation and the fMRI tasks (time 1), and after the fMRI tasks (time 2)**.

There was a significant main effect of fatigue condition (*F*_(1,27)_ = 16.87, *p* < 0.001) as well as a significant interaction between fatigue condition and time point (*F*_(2,54)_ = 5.30, *p* = 0.008). Follow-up paired sample *t*-tests showed that fatigue ratings were higher following the fatigue than the control manipulation at time 1 (*t*_(28)_ = 2.93, *p* = 0.007) and at time 2 (*t*_(28)_ = 5.32, *p* < 0.001). This indicates that the fatigue manipulation successfully induced greater feelings of fatigue in young and middle-aged participants compared to the control manipulation (Figure 1). Ratings did not differ significantly between young and middle-aged adults.

Significant differences were found between young and middle-aged adults in relation to feelings of fatigue associated with the teaching profession; middle-aged teachers (mean ratings = 69.7, 63.9, 38.3, standard deviation = 16.5, 20.2, 19.1 respectively) rated their job (*t*_(27)_ = 2.13, *p* = 0.043) and daily activities (*t*_(27)_ = 2.26, *p* = 0.032) as more fatiguing and indicated that fatigue interfered more with their ability to learn new things (*t*_(27)_ = 2.32, *p* = 0.028) than young teachers (mean ratings = 51.2, 45.6, 24.0, standard deviation = 29.0, 23.5, 13.4 respectively).

### Encoding and Recognition Task Behavioral Results

Regardless of age group or fatigue condition, participants correctly categorized between 92 and 95 of the 100 words presented during the encoding task. An average of 45–57 of these words were then subsequently recognized with high confidence on the recognition task (the proportions of subsequently recognized and subsequently forgotten, i.e., low confident hits as well as misses, words from the correctly categorized words are shown in Table 2).

**Table 2:** **Encoding task trials classified according to subsequent recognition task performance**.

	Young		Middle age	
	Control	Fatigue	Control	Fatigue
**Proportions from correctly categorized words**
Subsequently recognized	0.59 (0.16)	0.61 (0.12)	0.48 (0.22)	0.50 (0.22)
Subsequently forgotten	0.40 (0.16)	0.37 (0.10)	0.50 (0.21)	0.49 (0.20)
Reaction time (ms)
Subsequently recognized	1106 (149)	1106 (158)	1182 (124)	1187 (142)
Subsequently forgotten	1092 (125)	1087 (144)	1151 (141)	1145 (130)

*Mean (standard deviation)*.

First, the RT data were analyzed (see Table 2). An age group (young, middle age) × fatigue condition (fatigue, control) × subsequent recognition (high confident hits, forgotten words) repeated measures ANOVA revealed no effects of—nor interactions with—age group or fatigue condition. A main effect of subsequent recognition (*F*_(1,27)_ = 6.96, *p* = 0.014) indicated that, independent of age group and fatigue condition, responses to subsequently recognized words were slower than responses to subsequently forgotten words. This finding suggests that the encoding processes benefitted from more prolonged study of word stimuli (i.e., more prolonged study during the encoding task was more likely to result in subsequent high confidence recognition).

Table 3 displays the proportions of responses from the correctly categorized words distributed over the age groups and fatigue conditions. These data are used for the next analyses. Effects of age and fatigue on subsequent recognition task performance were investigated using an age group × fatigue condition × confidence (high, low) repeated measures ANOVA, with corrected recognition (i.e., the proportions of subsequently recognized minus false alarms) as dependent variable. This analysis revealed no effects of age or fatigue, but showed that high confidence corrected recognition scores were significantly higher than low confidence scores (*F*_(1,27)_ = 125.07, *p* < 0.001).

**Table 3:** **Recognition task performance**.

	Young		Middle age	
Response type	Control	Fatigue	Control	Fatigue
**High confidence**
Recognized	0.59 (0.16)	0.61 (0.12)	0.48 (0.22)	0.50 (0.22)
False alarms	0.15 (0.07)	0.18 (0.12)	0.09 (0.09)	0.10 (0.11)
Corrected recognition	0.44 (0.14)	0.43 (0.14)	0.39 (0.16)	0.40 (0.17)
**Low confidence**
Recognized	0.15 (0.11)	0.16 (0.10)	0.19 (0.11)	0.17 (0.07)
False alarms	0.16 (0.11)	0.17 (0.13)	0.13 (0.09)	0.12 (0.07)
Corrected recognition	0.01 (0.12)	0.02 (0.08)	0.06 (0.14)	0.05 (0.08)

*Mean (standard deviation) proportion of responses from the correctly categorized words. Corrected recognition = proportion of recognized minus false alarms. Note: since subsequently recognized words are defined as high confidence hits, the proportions of high confidence recognized words in this table match those in Table 2*.

We then conducted age group × fatigue condition × response type (subsequently recognized, false alarms) repeated measures ANOVAs per confidence level to investigate whether the responses represent reliable memory discrimination rather than guesses. Analysis of low confidence responses showed no significant difference between subsequently recognized words and false alarms (*p* = 0.256; also reflected in low confidence corrected recognition scores, which were essentially 0). This suggests that low confidence responses did not show memory above chance level. Analysis of high confidence responses, on the other hand, showed significantly more subsequently recognized words than false alarms (*F*_(1,27)_ = 276.72, *p* < 0.001), suggesting that high confidence subsequent recognition generally reflects successful memory encoding. This finding supports our fMRI contrast classification of encoding task words into subsequently recognized words (with high confidence only) and subsequently forgotten words (including low confidence subsequent recognition in addition to subsequently missed words). No effects of age or fatigue—or interactions with age or fatigue—were observed for either one of the confidence levels.

### Encoding Task fMRI Results

#### Task-Related Activation

Activation across both conditions associated with the successful encoding contrast in young and middle-aged adults was found in the ACC extending into the left dorsomedial PFC (DMPFC), the left ventrolateral PFC (VLPFC) extending into the orbital frontal cortex (OFC), and the left putamen (Table 4). This activation pattern is consistent with the major areas of activation evident in two meta-analyses examining successful encoding (Spaniol et al., 2009; Kim, 2011).

**Table 4:** **Task-related brain activation associated with successful encoding**.

			MNI coordinates		
Region		BA	*x*	*y*	*z*	*t*-value	Cluster size (voxels)
ACC/DMPFC	L	32/9	−9	39	39	3.81	38*
ACC	R	32	9	24	12	4.12	108*
	*R*	*33*	*3*	*15*	*18*	*3.45*	
VLPFC	L	6	−39	3	27	5.35	428*
*VLPFC*	*L*	*45*	*−51*	*27*	*12*	*5.27*	
*OFC*	*L*	*47*	*−45*	*39*	*−3*	*4.53*	
Putamen	L	–	−21	15	0	3.37	11*

Abbreviations: L, left, R, right, BA, Brodmann area, ACC, Anterior cingulate cortex, DMPFC, dorsomedial prefrontal cortex, VLPFC, Ventrolateral prefrontal cortex, OFC, Orbital frontal cortex, MNI, Montreal Neurological Institute space. Cluster level significance: *p_(FWE)_ < 0.05. Note: Italics are used to indicate additional activation peaks within a cluster.

#### Main Effect of Age

**Figure 2:**
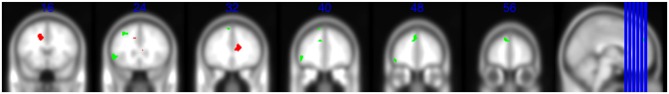
**A multislice view of activation effects in the prefrontal cortex (PFC) significant at *p*_(Family-wise error corrected)_ < 0.05, small volume corrected.** Locations of the slides are signified by the *y* coordinates, presented in blue at the top of the images. Green blobs illustrate the main effect of age group on successful memory encoding activation: greater dorsomedial, dorsolateral and orbital frontal PFC activation in middle-aged compared to young teachers. Red blobs illustrate the main effect of fatigue condition on successful encoding activation: decreased anterior cingulate cortex (ACC) activation in the fatigue compared to control condition (*y* = 16) as well as the interaction between group and fatigue condition: only the middle-aged teachers showed a significant reduction in activity in the fatigue condition compared to the control condition, and activation of this area in the control condition was higher in middle-aged compared to young teachers (*y* = 32).

A main effect of age was found, with middle-aged teachers showing greater successful encoding activation than young teachers in the left DMPFC, DLPFC and OFC (Table 5 and Figure 2).

**Table 5:** **Areas showing greater activation during successful encoding in middle-aged than in young teachers**.

			MNI coordinates		
Region		BA	*x*	*y*	*z*	*t*-value	p_FWE-SVC_
*Middle age > Young*
Dorsomedial PFC	L	9	−3	48	39	2.96	0.097
Dorsolateral PFC	L	9	−27	18	45	3.15	0.066
Orbital frontal	L	45	−48	42	−3	3.78	0.015
	L	45	−48	42	−9	3.61	0.023
	L	45	−45	48	−9	3.35	0.042

*Abbreviations: L, left, R, right, BA, Brodmann area, PFC, prefrontal cortex, MNI, Montreal Neurological Institute space, FWE-SVC, Family Wise Error-Small Volume Corrected*.

#### Main Effect of Fatigue Condition

A main effect of fatigue condition was found in the right ACC (peak coordinates *x* = 15 *y* = 33 *z* = 18; *t*-value = 3.62; *p*_FWE-small volume corrected (SVC)_ = 0.022), with greater activation evident in this area in the control than fatigue condition (Figure 2).

#### Age Group and Fatigue Condition Interaction

**Figure 3:**
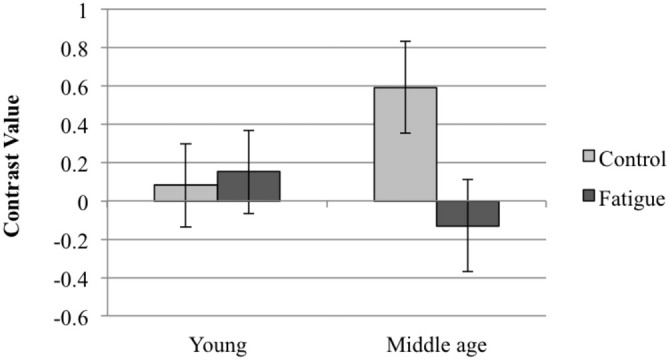
**The interaction between the effect of age group and fatigue condition on successful encoding activation in the left ACC (error bars show 90% confidence intervals)**.

A trend for an interaction was found in the left ACC (peak coordinates *x* = −15 *y* = 15 *z* = 45; *t*-value = 3.15; *p*_FWE−SVC_ = 0.065). In order to interpret the direction of this trend, we performed follow-up *t*-tests using the identified cluster as a ROI. The results indicated that activation in this cluster was greater in middle-aged than young adults in the control condition (*t*-value = 3.53, *p*_FWE−SVC_ = 0.028). Furthermore, middle-aged adults showed significantly reduced activation (*t*-value = 4.05, *p*_FWE−SVC_ = 0.008) in the fatigue compared to the control condition, whereas no significant activation change was evident in young adults (Figure 3).

#### *Post hoc* Correlations with Task Performance

Although corrected recognition scores showed equivalent performance in the two age groups, an examination of the number of high confidence subsequently recognized words alone revealed poorer performance in the middle-aged group (accompanied by greater variability on this measure in the middle-aged group than in the young group). Therefore, we also used regression analyses to examine the relationship between successful encoding-related activation and subsequent recognition task performance (using high confidence corrected recognition scores). We observed no significant interactions between age group or fatigue condition using ROI analyses within clusters identified as showing an age or fatigue related activation difference (i.e., left DMPFC, left DLPFC, left OFC and ACC clusters). We also examined correlations between RT to high confidence subsequently recognized words during the encoding task and brain activation, but we did not find significant (interaction) effects.

## Discussion

In the present study we examined the effects of age and induced cognitive fatigue on brain activation associated with the subsequent memory operationalization of successful encoding in young and middle-aged schoolteachers. This investigation resulted in a main effect of age group and a main effect of fatigue condition. Greater activation was found in middle-aged than young teachers in left and medial PFC areas. Secondly, the fatigue condition resulted in reduced activation compared to the control condition in the ACC.

### Age Group Differences

Behavioral performance on the neuropsychological WLT test and the association with subsequent recognition performance on the fMRI task reflected the typical pattern of aging effects on memory; memory recall showed signs of age-related cognitive decline in middle-aged teachers, whereas recognition performance remained relatively unaffected (Yonelinas, 2002). Furthermore, scores on the Dutch adult reading test, a measure of crystallized intelligence, showed the expected increase with age.

As expected, significant brain activation differences between young and middle-aged teachers were found to underlie these subtle differences at the behavioral level, which is consistent with subtle changes found in earlier studies (Jacobs et al., 2013; Klaassen et al., 2013, 2014). Our findings demonstrated the presence of similar age-related activation differences between young and middle-aged teachers to those found in previous studies comparing young and old adults (Morcom et al., 2003; Gutchess et al., 2005; Dennis et al., 2007). In line with the CRUNCH hypothesis (Reuter-Lorenz and Cappell, 2008), middle-aged teachers showed increased PFC activation, which in the absence of performance difference may be an indication of neural compensation. For instance, the left DLPFC was found to be commonly over-recruited in older adults (Maillet and Rajah, 2014). The age-related differences in our middle-aged group were less extensive than those found by previous studies in older adults and there are no indications for declines elsewhere (which could have accounted for the necessity of neural compensation).

No correlations were found between the increased left PFC activation in middle-aged compared to young adults and task performance. Furthermore, the age-related increase in PFC activation was not paired with an age-related decrease in MTL activation. As such there is little indication that the increase in left PFC activation in middle-aged compared to young adults fulfilled a neural compensatory function. Dennis et al. (2007) suggested that a similar increase in left PFC activation in older adults in relation to successful encoding might reflect deeper or more elaborate semantic processing. On the basis of a meta-analysis examining age-related differences across multiple cognitive domains, Spreng et al. (2010) suggested that increased left PFC activation in older adults might be attributable to a greater reliance on strategic control. Either or both of these explanations may therefore also account for increased left PFC activation in middle-aged adults in the current study.

It should be noted that while cross-sectional analyses have shown over-recruitment of frontal areas with increasing age, longitudinal analyses have shown under-recruitment of frontal areas with age. This discrepancy has been attributed to the effects of high performing older adults, such as those included in the present study, on cross-sectional findings. When followed longitudinally, these higher performing older adults also demonstrated a reduction in brain activation with age (Nyberg et al., 2010). As such, although our high performing middle-aged adults currently show relative over-activation compared to the younger group, they may show reductions in brain activation with age (following or in conjunction with greater bilateral recruitment) when followed longitudinally into older age. Future longitudinal studies following participants who are young adults to middle age and to old age are essential for the advancement of our understanding of brain activation changes across the lifespan.

### Fatigue Condition Effects

Subjective fatigue ratings were higher in all participants in the fatigue compared to the control condition, indicating that the fatigue manipulation successfully induced a state of increased fatigue. As expected, the fatigue condition was associated with a decrease in brain activation, indicating a fatigue-related depletion of cognitive resources. This decrease in activation was not evident in the hypothesized left PFC, but it was evident in the hypothesized ACC. As such, left PFC processes which we suggested play a role in the cognitive control of memory in particular may have been less affected by fatigue than the more general cognitive control processes attributed to the ACC.

In an event-related potential study, Lorist et al. (2005) also reported changes in ACC activity, as indicated by a reduction in the error-related negativity (ERN) response in young adults as a result of induced cognitive fatigue. The authors attributed this finding to impaired cognitive control, resulting in compromised error monitoring and inadequate performance adjustment. ACC activation is generally associated with response monitoring, adjustment to errors and correct performance on tasks with high-conflict trials (e.g., Botvinick et al., 2004). In light of this, higher ACC activation would indicate higher evaluative control, which would logically lead to higher task performance. Conversely, the present finding showed no relation between ACC activation and cognitive task performance. It has been suggested that activation differences in the ACC may reflect the role of this area in energizing task-related areas that lack resources due to fatigue (Vallesi, 2012). However, as our fatigue condition was associated with a decrease in ACC activation and no associated decrease in other areas, an energizing role was not apparent.

It is remarkable that we observed a reduction in ACC activation in response to the fatigue manipulation, whereas no changes in behavioral performance were observed. This may relate to the notion that neuroimaging data can be more sensitive than behavioral data, which is more often observed (e.g., Veroude et al., 2013). It may also indicate that this area—even though the ACC has been related to changes in fatigue state, as outlined above—corresponds to processes that are not vital to encoding success *per se*. It is also remarkable that we observed neural activation changes in response to the fatigue manipulation in both groups. This suggests that the ACC may play a role in the experience of (mental) fatigue in general and not just in the aging brain. Involvement of the ACC in fatigue has previously been suggested based on neuroimaging studies in clinical populations (e.g., patients with multiple sclerosis or chronic fatigue syndrome; DeLuca et al., 2009; Dobryakova et al., 2013), which aligns well with the present results. As suggested by Dobryakova and colleagues, changes in ACC activation could indicate a difference in effort. Whether this area may be similarly involved in induced fatigue in healthy populations remains to be elucidated.

With respect to the hypothesized differential effects of the fatigue condition in young and middle-aged adults, we observed no significant interaction. It is worth mentioning, however, that there was a trend (*p* = 0.065) for an interaction between age group and fatigue condition in the left ACC. This trend followed the direction of our hypothesis. This near-significant interaction is consistent with additional activation decreases in middle-aged compared to young adults. As such, this is consistent with greater resource depletion in the middle-aged group. It is relevant in this respect that the middle-aged teachers showed greater activation of this area in the control condition than the young teachers. In light of the CRUNCH hypothesis (Reuter-Lorenz and Cappell, 2008) this may indicate neural compensation to maintain performance at an equivalent level. The greater reduction in response to the fatigue induction may indicate that, due to increased compensation, a so-called resource ceiling is reached earlier in the middle-aged teachers compared to the young. This is also in line with CRUNCH. Yet, according to this theory, the reduction in brain activation should be accompanied by a decline in performance, which was not observed in the present study. The findings obtained here should be interpreted with caution, as it is a trend that did not reach statistical significance. The findings do suggest that it is relevant to extend this research in order to evaluate the possible differential responses to a fatigue induction in relation to age. This is especially the case because the present findings were done in middle-aged teachers who were relatively young.

### Strengths and Limitations

Interestingly, despite equivalent education level and occupation type in the two age groups, middle-aged teachers were characterized by significantly higher letter fluency scores. This finding suggests higher verbal intelligence in the middle-aged group, as letter fluency has been shown to decline with age (van der Elst et al., 2006b). However, since letter fluency did not correlate with performance on the fMRI task, we consider it unlikely that this difference significantly influenced fMRI findings. Nevertheless, it should be taken into account that the groups may differ with regard to aspects other than age, such as years of experience and job responsibilities (e.g., middle-aged teachers may have more senior jobs that are more taxing). We also note that, although the fatigue condition provides greater insight into age-related differences by examining them in the context of induced fatigue, a factor encountered during the workday, future studies may extend the ecological validity of this investigation to more real-world conditions, such as fatigue induced by a real-life workday. Furthermore, although we chose to focus on males in order to minimize unwanted variance in our small sample group, future studies should consider examining these effects in larger samples including females.

A strength of the present study is that cognitive aging was investigated in a homogeneous population of working adults — male schoolteachers —, rather than using the typical comparison between young university students and retired older adults, often utilized in aging studies. This comparison minimized many of the drawbacks of using a cross-sectional design to investigate age-related changes in brain activation (as, for example, significant differences in working memory task performance between the two age groups were not evident; also see Burgmans et al., 2010 for structural differences between young and older-aged individuals). The homogeneous population of male schoolteachers was also advantageous in relation to the investigation of induced cognitive fatigue effects, providing relative homogeneity in relation to experience with cognitively demanding tasks and education level.

In conclusion, the findings from the present study suggest that middle-aged teachers recruit greater neural resources than younger teachers in relation to similar levels of cognitive performance. The present findings need replication in order to confirm that cognitive resources in middle-aged schoolteachers are depleted more than in young teachers in a situation of sustained performance of cognitively demanding tasks. The findings may have relevance for school and teaching practice in that they provide insight into the increased cognitive effort and the resulting fatigue, which quite some middle-aged teachers experience in the course of their work. Recognizing the strengths and weaknesses of middle-aged teachers (and perhaps adjusting performance expectations and support strategies accordingly) is not only important in order to enhance workplace performance, it is probably also an important step towards addressing the relatively high incidence of burnout in the teaching profession, notably in the middle-aged teacher (Hakanen et al., 2006).

Investigating cognitive aging effects in middle-aged adults in the context of challenging factors encountered during the workday, such as the sustained performance of cognitively demanding tasks, is an important step towards a better understanding of the real-life impact of cognitive decline in this age group. Furthermore, findings from the present study provide insight into possible mechanisms underlying age-related differences in teachers’ professional performance and work-related fatigue complaints, and thus indicate novel avenues for research into interventions to improve workplace performance and satisfaction.

## Author Contributions

EKB collected, processed, analyzed and interpreted the data. The manuscript was prepared by EKB with contributions from all co-authors. SP revised and adjusted the manuscript, and performed analyses in accordance with the reviewers’ suggestions. EATE collected data and advised on data processing, analysis and interpretation. RHMdG contributed to participant recruitment and project management. WHB provided support with MRI scanning protocols and data processing. DJV contributed to data processing, analysis and interpretation. JJ initiated the study, managed scientific progress, was responsible for interpretation of the findings and revised and adjusted the manuscript.

## Funding

The research described in this article was funded by the Dutch Science Organization NWO under grant number 056-35-011.

## Conflict of Interest Statement

The authors declare that the research was conducted in the absence of any commercial or financial relationships that could be construed as a potential conflict of interest.
